# Evidence that the Bowman-Birk inhibitor from *Pisum sativum* affects intestinal proteolytic activities in chickens

**DOI:** 10.1016/j.psj.2023.103182

**Published:** 2023-10-11

**Authors:** Thierry Moreau, Emilie Recoules, Marion De Pauw, Valérie Labas, Sophie Réhault-Godbert

**Affiliations:** ⁎ INRAE, University of Tours, BOA, 37380 Nouzilly, France; † INRAE, CNRS, IFCE, University of Tours, PRC, 37380 Nouzilly, France; ‡ INRAE, CHU of Tours, University of Tours, PIXANIM, 37380 Nouzilly, France

**Keywords:** chicken, pea, digestion, inhibitor, protease

## Abstract

Chicken diet essentially relies on soybean as the major source of proteins but there are increasing efforts to identify other protein-rich feedstuffs. Of these, some pea cultivars constitute interesting sources of proteins, although some of them contain antinutritional factors that may compromise the digestibility of their protein content. Consequently, chickens exhibit low performance, while undigested compounds rejected in feces have a negative environmental impact. In this article, we analyzed the intestinal content of chickens fed a pea diet (*Pisum sativum*) to decipher the mechanisms that could explain such a low digestibility. Using gelatin zymography, we observed that the contents of chicken fed the pea diet exhibit altered proteolytic activities compared with intestinal contents from chickens fed a rapeseed, corn, or soybean diet. This pea-specific profile parallels the presence of a 34 kDa protein band that resists proteolysis during the digestion process. Using mass spectrometry analysis, we demonstrated that this band contains the pea-derived Bowman-Birk protease inhibitor (**BBI**) and 3 chicken proteases, the well-known chymotrypsinogen 2-like (**CTRB2**) and trypsin II-P39 (**PRSS2**), and the yet uncharacterized trypsin I-P38 (**PRSS3**). All 3 proteases are assumed to be protease targets of BBI. Molecular modeling of the interaction of pea BBI with PRSS2 and PRSS3 trypsins reveals that electrostatic features of PRSS3 may favor the formation of a BBI-PRSS3 complex at physiological pH. We hypothesize that PRSS3 is specifically expressed and secreted in the intestinal lumen to form a complex with BBI, thereby limiting its inhibitory effects on PRSS2 and chymotrypsinogen 2-like proteases. These data clearly demonstrate that in chickens, feedstuff containing active pea BBI affects intestinal proteolytic activities. Further studies on the effects of BBI on the expression of PRSS3 by digestive segments will be useful to better appreciate the impact of pea on intestine physiology and function. From these results, we suggest that PRSS3 protease may represent an interesting biomarker of digestive disorders in chickens, similar to human PRSS3 that has been associated with gut pathologies.

## INTRODUCTION

Soybean is the first protein-rich feedstuff used by the poultry sector. The other sources of plant proteins for poultry nutrition include rapeseed, chickpeas, sunflowers seeds, etc. (Iji, et al., 2017). However, as some of these legumes and oilseeds are mainly imported from North and South America, and China (Iji, et al., 2017), many countries are exploring alternatives that are produced locally to reduce carbon footprint. Of these alternatives, pea is an interesting protein source that can be used as an alternative to soybean, especially in organic livestock farming where genetically modified soybean and inclusion of industrial amino acids in the feed are prohibited (Schumacher et al., 2011). However, pea contains several antinutritional factors including protease inhibitors, tannins, lectins, and phytate (Savage, 1989) that reduce the overall digestibility of its protein content and consequently decrease the growth performance of broilers (Velayudhan et al., 2019; Toghyani et al., 2020). To increase pea digestibility, several strategies may be used. Indeed, some of these antinutritional factors may be partially inactivated by various industrial treatments including steam pelleting (Carré et al., 1987), hydrothermal treatments (Urbano et al., 2003), extrusion (Alonso et al., 2000; Hejdysz et al., 2016), micronization, and dehulling processes (Igbasan and Guenter, 1996). Other strategies to overcome the poor digestibility of plant-based feedstuffs are the inclusion of exogenous enzymes such as phytase (Urbano et al., 2003), exogenous proteases (Hejdysz et al., 2020; Szczurek and Świątkiewicz, 2020), and carbohydrases (Cowieson et al., 2003). The selection of pea cultivars containing natural BBI mutants with reduced or very low inhibitory activity has also been investigated (Clemente et al., 2015).

The underlying mechanisms of pea low digestibility is still not completely understood but there is increasing literature demonstrating that pea components when consumed, modify the physiology and thereby the function of the digestive tract. These pea antinutritional factors have been reported to be associated with mucosal immune responses in jejunum of broilers (Röhe et al., 2017). In calves, pancreas size and enzyme activities were shown to increase with the pea diet compared with soybean diet (Le Dréan et al., 1995). Although a pea diet does not seem to modify the intestinal microflora of broilers, the count for some bacteria (enterococci in the small intestine and *Clostridium perfringens* and coliforms in the caeca) was shown to be higher compared with the control group (maize diet) (Brenes et al., 1989).

Recently, we have published some proteomic data on the jejunal contents of broilers fed 4 different diets containing soybean meal, rapeseed meal, pea or corn distiller's dried grain with solubles, as the only protein source (Recoules et al., 2017). Our previous results revealed the presence of 4 undigested pea proteins in the jejunal content: a trypsin inhibitor (Bowman-Birk inhibitor, **BBI**), convicilin (gi:7339551), legumin A2 (gi: 126161), and the pea lectin (gi:4389007) that are all known antinutritional factors. Intriguingly, a chicken protease (PRSS3) was specifically identified in the samples from the broilers fed a pea diet (Recoules et al., 2017). The concomitant presence of pea protease inhibitor and a newly identified trypsin-like protease (PRSS3) in the digestive contents of chicken prompted us to investigate the molecular mechanisms that accompany the proteolytic digestion of pea-enriched feedstuff.

Thus, the objective of this research was to explore whether the BBI contained in the pea diet is active, and whether it can affect the activity of digestive enzymes in chickens. The samples used were from the experiment aforementioned (Recoules et al., 2017). They were analyzed by gelatin zymography to visualize proteolytic activities, and undigested proteins were identified by SDS-PAGE followed by mass spectrometry. In parallel, 2 proteoforms of BBI purified from the pea diet by trypsin-affinity and exclusion chromatography, were identified by mass spectrometry and their inhibitory activities were assessed using enzymatic assays. The interaction of chicken trypsin-like proteases with active BBI was investigated in silico by analyzing the physicochemical features associated with their respective 3D structures. Altogether, these data bring new insights in the dynamics of digestive trypsin-like proteases in response to diet-derived BBI, in broilers.

## MATERIALS AND METHODS

### Animal Sample Collection

Digestive contents were obtained from an experiment that was published previously (Recoules et al., 2017), and no additional experiments on animals were required for this study. Briefly, from d 7 to d 17, Ross PM3 broiler chicks (Grelier, Saint-Laurent-de-la-Plaine, France) were fed semisynthetic experimental diets (50/50 mix with the starter diet) where the protein fraction was based on a single protein source: soybean meal, rapeseed meal, pea, or corn distiller's dried grains with solubles. Between d 17 and 21, chickens were fed the experimental diet. On d 21, chickens were euthanized and contents from the different parts of the digestive tract (proventriculus/gizzard, duodenum, jejunum, and ileum) were collected. As mentioned in the article (Recoules et al., 2017), the experimental procedures involving the use of chicken were initially approved by the regional Ethics Committee (Approval No. C37-175-1). All experiments were conducted according to the European legislation on the “protection of animals used for experimental and other scientific purposes” set by the European community Council Directive of November 24, 1986 (86/609/ECC).

### Zymography and SDS-PAGE Analysis and of Digestive Contents

Crude digests from proventriculus/gizzard, duodenum, jejunum, and ileum, obtained after feeding chickens with a diet composed of pea, soybean, corn, or rapeseed (12 animals per diet), were grounded in 0.5 M Tris-HCl buffer (pH 8.8 (diet) or 6.8 (digests), 150 mM NaCl) using a T25 Ultra Turrax (IKA, Staufen, Germany). Resulting samples were centrifuged to pellet and eliminate insoluble components. The protein concentration of supernatants was determined using the DC-Biorad Assay (Bio-Rad, Marnes-la-Coquette, France), and bovine serum albumin (Interchim, Montluçon, France) as the standard. Pools per digestive segment per diet were produced, and resulting samples were stored at −20°C until further use (gelatin zymography and SDS-PAGE under nonreducing/nonboiling conditions).

Gelatinolytic activities were assessed, as previously described (Réhault-Godbert et al., 2008). Briefly, 4 µg of proteins were diluted in 5× Laemmli buffer under denaturing but nonreducing conditions. Samples were not boiled to preserve protease activity, as recommended for zymography studies. Samples were loaded onto a 12.5% acrylamide-bis acrylamide gel containing 0.3 mg/mL gelatin, and separated by electrophoresis. Protein renaturation was achieved by soaking gels in 2.5% Triton X-100 for 1 h (2 × 30 min) at room temperature under constant agitation. Gels were then incubated for 1 h at 41°C (physiological temperature of chickens) in activation buffer (0.5 M Tris-HCl, pH 6.8), followed by Coomassie Blue staining. The proteolytic degradation of gelatin by protease(s) was visualized as clear zones on a blue background (white zones on a black background in the corresponding figures). Stained gels were scanned using an Odyssey IR fluorescence imaging system (Li-Cor Biosciences, Lincoln, NE) (Harris et al., 2007).

### Identification of Proteins Composing the 34 kDa Band (Pea Diet)

Parallel to zymography, 4 µg of samples were loaded onto a 12.5% acrylamide gel without gelatin. After fractionation, gels were stained with Coomassie Blue and the 34 kDa band of proteins was excised. The resulting gel pieces were washed in water: acetonitrile solution (v/v: 1:1, 5 min) followed by 100% acetonitrile (10 min). Reduction and cysteine alkylation was performed by successive incubation with 10 mM dithiothreitol in 50 mM NH_4_HCO_3_ (30 min, 56°C), then 55 mM iodoacetamide in 50 mM NH_4_HCO_3_ (20 min, room temperature, in dark). Pieces were then incubated with 50 mM NH_4_HCO_3_ and acetonitrile (1:1, 10 min) followed by acetonitrile (15 min). In-gel digestion was carried out overnight using 25 mM NH_4_HCO_3_ with 12.5 ng/μL bovine trypsin (Roche Diagnostics GmbH, Mannheim, Germany). Resultant peptides were extracted by successive incubation in 5% formic acid, followed by acetonitrile and 1% formic acid (1:1, 10 min), and 100% acetonitrile (5 min). For all peptide extractions, supernatants were pooled and dried using a SPD1010 speedvac system (Thermosavant, Thermo Fisher Scientific, Bremen, Germany). The resulting peptide mixture was analyzed by nanoflow liquid chromatography tandem mass spectrometry (NanoLC-MS/MS). All experiments were performed on a dual linear ion trap Fourier transform mass spectrometer (FT-MS) LTQ Orbitrap Velos coupled to an Ultimate 3000 RSLC Ultra High Pressure Liquid Chromatographer, controlled by Chromeleon Software (version 6.8 SR11) (Thermo Fisher Scientific, Bremen, Germany). Samples were desalted and concentrated for 10 min at 5 µL/min on an LCPackings trap column (Acclaim PepMap 100, C18, 75 µm inner diameter × 2 cm long, 3 µm particles, 100 Å pores). The peptide separation was conducted using a LCPackings nano-column (Acclaim PepMap C18, 75 µm inner diameter × 50 cm long, 2 µm particles, 100 Å pores) at 300 nL/min, by applying linear gradient consisted of 4 to 60% B during 90 min. Mobile phases consisted of (A) 0.1% formic acid, 97.9% water, 2% acetonitrile (v/v/v), and (B) 0.1% formic acid, 15.9% water, 84% acetonitrile (v/v/v). Data were acquired in positive mode in data-dependent mode to automatically switch between high resolution full-scan MS1 spectra (R at 60,000) and low resolution CID-MS2, in the 300 to 1,800 *m*/*z* range. The 20 most intense peptide ions with charge states ≥ 2 were sequentially isolated and fragmented in the linear ion trap using CID mode (collision energy 35%, activation time 10 ms, Qz 0.25). Dynamic exclusion was activated during 30 s with a repeat count of 1. The lock mass was enabled for accurate mass measurements. Polydimethylcyclosiloxane (*m*/*z*, 445.1200025 (Si(CH_3_)_2_O)_6_) ion was used for internal recalibration of the mass spectra. MS/MS ion searches were performed using Mascot search engine version 2.3.2 (Matrix Science, London, UK) via Proteome Discoverer 2.1 software (Thermo Fisher Scientific, Bremen, Germany) against NCBIprot_viridiplantae or NCBIprot_chordata database (January 2017). The search parameters included trypsin as a protease with 2 allowed missed cleavages and carbamidomethylcysteine, methionine oxidation and acetylation of N-term protein as variable modifications. The tolerance of the ions was set to 5 ppm for parent and 0.8 Da for-fragment ion matches. Peptides and proteins identified by MASCOT were validated using “Peptid Prophet” and “Protein Prophet” algorithm with Scaffold software (version 4.8.4, Proteome Software, Portland). Protein identifications were accepted if they contained at least 2 identified peptides.

### Purification of BBI From Pea Diet

The purification of BBI was performed using the pea diet as the starting material. The use of the pea diet was preferred to the crude pea powder, to take into account possible interaction of pea proteins with the other components composing the diet (fibers, other proteins, etc.). The experimental diet with pea as the protein source is available in (Recoules et al., 2017). The diet was composed of pea (850 g/kg), corn starch (57.9 g/kg), sucrose (28.9 g/kg), soybean oil (30 g/kg), dicalcium phosphate (15 g/kg), calcium carbonate (10 g/kg) salt (3), vitamin mineral premix (5 g/kg), and clinacox anticoccidian (0.2 g/kg). Trypsin inhibitory activity (likely due to BBI) was 6.6 International Trypsin Unit/mg (**ITU**).

Pea diet (10 g) was dissolved in 100 mL of 0.5 M Tris-HCl, 150 mM NaCl, pH 8.8, was grounded using T25 Ultra Turrax, as described above, and centrifuged during 15 min at 4,500 × *g*, 4°C. Supernatants were collected and protein concentration was estimated using absorbance at 280 nm (Nanodrop, ND-1000 spectrophotometer, Thermo Fischer Scientific, MA). The resulting supernatant was incubated with 2 mL of trypsin-agarose chromatography gel (Sigma-Aldrich, Saint-Louis, MO) that was previously equilibrated with 50 mM Tris-HCl, 150 mM NaCl, pH 7.4, and loaded onto a polypropylene column (Qiagen, Hilden, Germany). After extensive washes with 50 mM Tris-HCl, 150 mM NaCl, pH 7.4, bound proteins were eluted using 100 mM glycine-HCl, 0.5 M NaCl, pH 2.0 and samples were immediately neutralized with 1 M Tris-HCl to avoid protein denaturation. About 2 mg of proteins (including BBI) could be recovered and was subjected to a size exclusion chromatography (HighLoad 16/600 Superdex 75 pg prepacked column, GE Healthcare, Chicago, IL), equilibrated with 50 mM Tris-HCl, 300 mM NaCl, pH 7.4. The 2 major HPLC peaks (exhibiting major bands between 10 and 15 kDa on a 15% SDS-PAGE) were analyzed by MALDI-TOF mass spectrometry in order to measure intact masses of the biomolecules. The HPLC fractions were desalted and concentrated using zip tip C4 (Millipore, Guyancourt, France) and dried using a SPD1010 speedvac system (Thermosavant, Thermo Fisher Scientific, Bremen, Germany). Biomolecules were dissolved in 4 µL of 1% formic acid. Onto a MTP Polished 384 MALDI plate (Bruker Daltonics, Germany), using the dried droplet method, 1 µL of sample was overlaid with 1 μL of freshly prepared saturated α-cyano-4-hydroxycinnamic acid (**CHCA**) matrix dissolved in 50% acetonitrile/ 50% water in presence of 0.1% trifluoroacetic acid (**TFA**). The matrix/sample mix was allowed to evaporate at room temperature for 30 min before MALDI-MS analysis. Spectra were acquired using a Bruker UltrafleXtreme MALDI-TOF instrument (Bruker Daltonics, Bremen, Germany) equipped with a Smartbeam laser at 2 kHz laser repetition rate following an automated method controlled by FlexControl 3.0 software (Bruker Daltonics, Bremen, Germany). Spectra were obtained in positive linear ion mode in the 2,000 to 30,000 *m*/*z* (mass/charge). External calibration was performed using 1 μL of calibration solution containing Glu1-fibrinopeptide B, adreno corticotropic hormone (fragment 18–39), insulin, ubiquitin, cytochrome C, myoglobin, and trypsinogen. After external calibration, each spectrum was collected as a sum of 1,000 laser shots in 5 shot steps (5,000 spectra) with a laser parameter set at medium. MALDI spectra annotations were performed using the UniprotKB Seed trypsin/chymotrypsin inhibitor IVB sequence for *Pisum sativum* (P56679 entry IBBB_PEA) considering the 7 disulfide bridges known as modifications. These mass annotations were confirmed by a protein identification using bottom-up proteomics.

### Inhibitory Activities of BBI Proteoforms

Bovine trypsin, bovine chymotrypsin, Tosyl (**Tos**)-EGR-pNA, and Suc-AAPF-paranitroanilide (**pNA**) were from Sigma-Aldrich (Saint-Quentin Fallavier, France). Assays were performed in 96-well microplates using Tecan Infinite M200 microplate reader (Tecan France SAS, Lyon, France). Antitrypsin activity of BBI (peak 1 and peak 2) was assessed by incubating trypsin (8 nM), peak 1 or peak 2 (2, 10 nM) in 50 mM Tris, 0.15 M NaCl pH 7.4 for 10 min at 37°C prior to addition of Tos-EGR-pNA substrate (0.6 mM final). Absorbance was measured at 410 nm during 20 min. Hydrolysis rate was compared to that of the control consisting of trypsin (8 nM) and Tos-EGR-pNA substrate (0.6 mM). Antichymotrypsin activity was studied, as described above, except that we used chymotrypsin as the enzyme (10 nM), peaks 1 and 2 (2.5 and 12 nM), and succinyl (**Suc**)-AAPF-pNA (1.2 mM) as the substrate. The control consisted of chymotrypsin (10 nM) and Suc-AAPF-pNA (1.2 mM). Absorbance was read at 410 nm for 20 min.

### Molecular Modeling of Chicken Proteases PRSS2 and PRSS3 Complexed to BBI

A 3D model of each chicken protease was built by comparative modeling using SWISS-MODEL software (Waterhouse et al., 2018) at Expasy portal (www.expasy.ch) and the mature sequence of the enzyme (UniprotKB accession codes Q90628 and Q90629 for PRSS2 and PRSS3, respectively). In the automated modeling procedure, porcine trypsin (Protein Data Bank (**PDB**) accession code: 3MYW) was used as a template to generate chicken PRSS2 model. Similarly, bovine beta trypsin (PDB accession code: 1G3B) was used as a template to model chicken PRSS3 as both proteases share 75% protein sequence identity. Because the sequence identity of chicken proteases with their respective reference template is quite high, the modeling procedure was straightforward and the quality of the models was found to be satisfactory, as confirmed by the quality analysis performed by SWISS-MODEL tool.

The complex of each chicken protease with pea BBI was built using the X-ray coordinates of the bovine trypsin/soybean BBI complex (PDB accession code: 1D6R) and the X-ray structure of pea BBI (PDB accession code: 1PBI). To build each protease/inhibitor complex, PRSS2 or PRSS3 models and pea BBI were superimposed onto the corresponding protease and inhibitor of the trypsin/soybean BBI complex. The superimposition was performed by structural alignment using the minimum root mean square deviation (**r.m.s.d**) method. Pea BBI structure is a crystallographic dimer in which the carboxy-tail moiety of each BBI molecule adopts an extended conformation that stabilizes the dimer through the formation of a beta sheet-like structure (Li de la Sierra et al., 1999). Since it is unlikely that this dimer represents the functional state of BBI (Li de la Sierra et al., 1999), the carboxy-terminus from Leu67 to Asn73 was removed in the BBI monomer used to build the protease/BBI complex. Values of r.m.s.d after superimposition were 1.52 Å, 0.48 Å, and 0.33 Å for PRSS2-trypsin, PRSS3-trypsin, and pea BBI-soybean BBI pairs, respectively. These results indicate a high structural similarity of the reconstructed complexes with the trypsin/soybean BBI complex. A quick minimization using Swiss-PDBViewer (Guex and Peitsch, 1997) was performed to overcome minor steric constraints at the interface between PRSS2 or PRSS3 protease, and pea BBI.

Electrostatic calculations were performed using the Pymol software (Schrödinger, 2021) and Adaptive Poisson-Boltzmann Solver plugin (Baker et al., 2001). The color-coded electrostatic potential molecular surface was represented using Pymol, using a conventional color scheme ranging from red (negatively charged regions) to deep blue (positively charged regions).

## RESULTS

### Digestive Proteolytic Profile of Chickens Fed the Pea Diet Differs Significantly From Chickens Fed Another Protein Source

**Figure 1:**
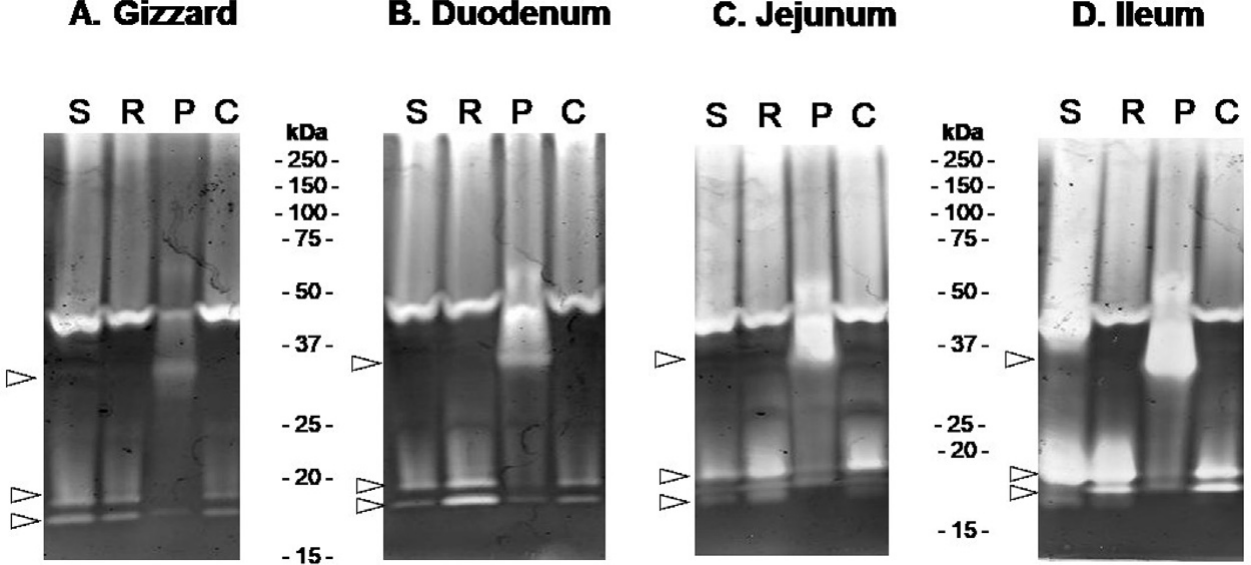
Gelatinolytic activities of secretions along the chicken gastro-intestinal tract. (A) Gizzard; (B) Duodenum; (C) Jejunum; (D) Ileum. Proteolytic activities were assessed using a pool of 12 chicken digestive contents per digestive segment and diet. The homogeneity of each individual sample was initially assessed by SDS-PAGE, prior to pooling. S, soybean; R, rapeseed; P, pea; C, corn. White arrows indicate bands that differ in intensity in the pea diet experiment.

Proteolytic activities of digestive contents (gizzard, duodenum, jejunum, and ileum) sampled from chicken fed soybean, pea, rapeseed, and corn were assessed by gelatin zymography under nonreducing conditions. Samples corresponding to the chickens fed the pea diet exhibit a very specific profile, while samples from the chickens fed the other protein source are comparable (Figure 1). This pea diet specificity is detectable regardless of the digestive segment. Strikingly, the intensity of the signal corresponding to a band of 34 kDa apparent molecular mass is increasing progressively from the gizzard to the ileum.

### A 34 kDa SDS-PAGE Band Exhibiting Gelatinolytic Activities Contains Bowman-Birk Inhibitor and Chicken Digestive Proteases

**Figure 2:**
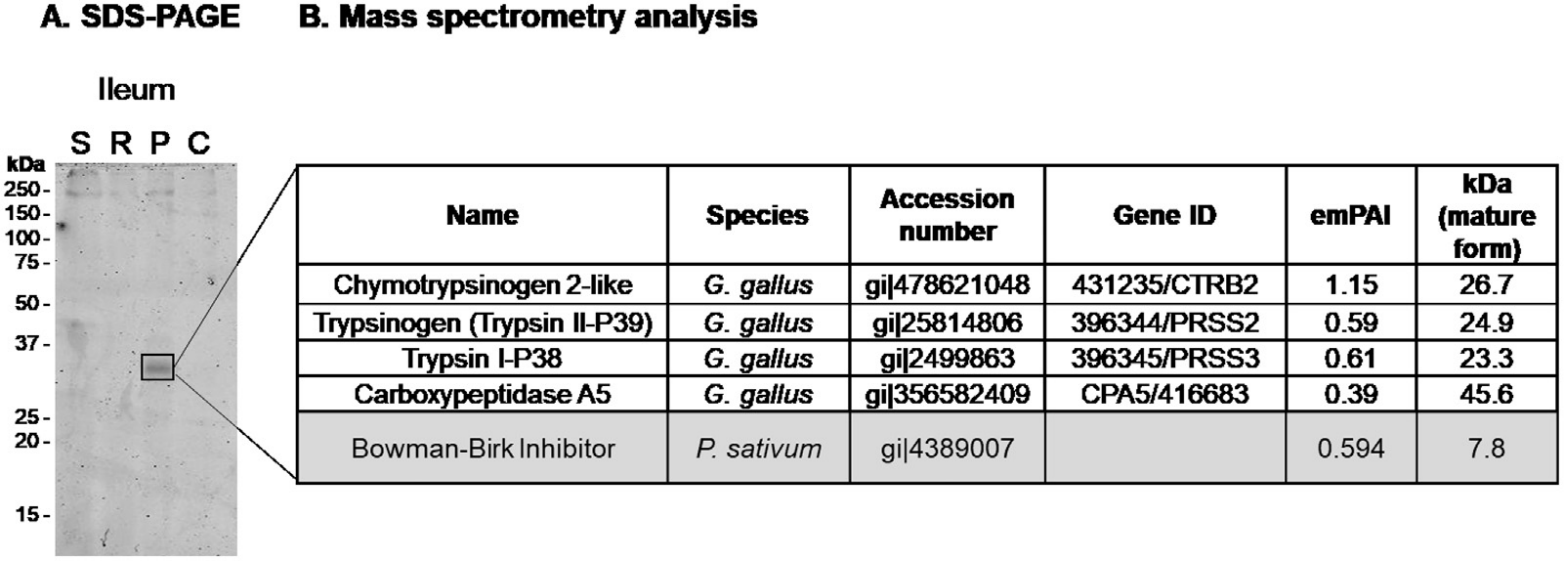
Identification of proteins composing the 34 kDa band from the pea diet. The SDS-PAGE band of 34 kDa was cut (A) and further analyzed by mass spectrometry (B), as described in Materials and Methods section.

The ileal content from chickens fed the 4 protein diets was analyzed by SDS-PAGE under nonreducing conditions (Figure 2A). The profile corresponding to the pea diet exhibits a 34 kDa band that is not detectable in the other samples. This 34 kDa band seems to correspond to the 34 kDa band detected in the zymography gel (Figure 1). Mass spectrometry analysis of this band revealed the presence of Bowman-Birk inhibitor from pea seed (BBI, gi: 4389007) and 4 chicken proteases: chymotrypsinogen 2-like (gene ID: 431235/CTRB2), trypsinogen (gene ID: 396344, PRSS2), trypsin I-P38 (gene ID: 396345, PRSS3), and carboxypeptidase A5 (gene ID: 416683, **CPA5**) (Figure 2B). Chymotrypsinogen 2-like precursor was identified with 6 peptides (TTDTVVLGEYDQETASSDVQR, LGIAKVFR, VFRNPSYSSLTIR, NPSYSSLTIR, LATPAQLNAR, LREWIDSVLAAN), trypsin II-P29 was identified with 2 peptides (LGEYNIDVQEDSEVVR, LASAVEYSADIQPIALPSSCAK), trypsin I-P38 was identified with 3 peptides (LGEYNLAAQDGSEQTISSSK, LGEYNLAAQDGSEQTISSSKVIR, VCNYVSWIK), and carboxypeptidase A5 with 5 peptides (EWVTQ-ATGVWTANK, FSTGGSNRPAVWLDTGIHSR, NWDAGFGGSGSSSNPCSETYHGPYAHSESEVK, SIHAGSSCIGVDPNR, SIVDFIQSHGNVK). Using *Pisum sativum* as the reference database, BBI was identified with 2 exclusive, unique peptides (GDDVKSACCD, CQCFDTQKFCYK), which correspond to 39% sequence coverage.

### Characterization of the Trypsin-Inhibitor From the Pea Diet (Bowman-Birk Inhibitor)

**Figure 3:**
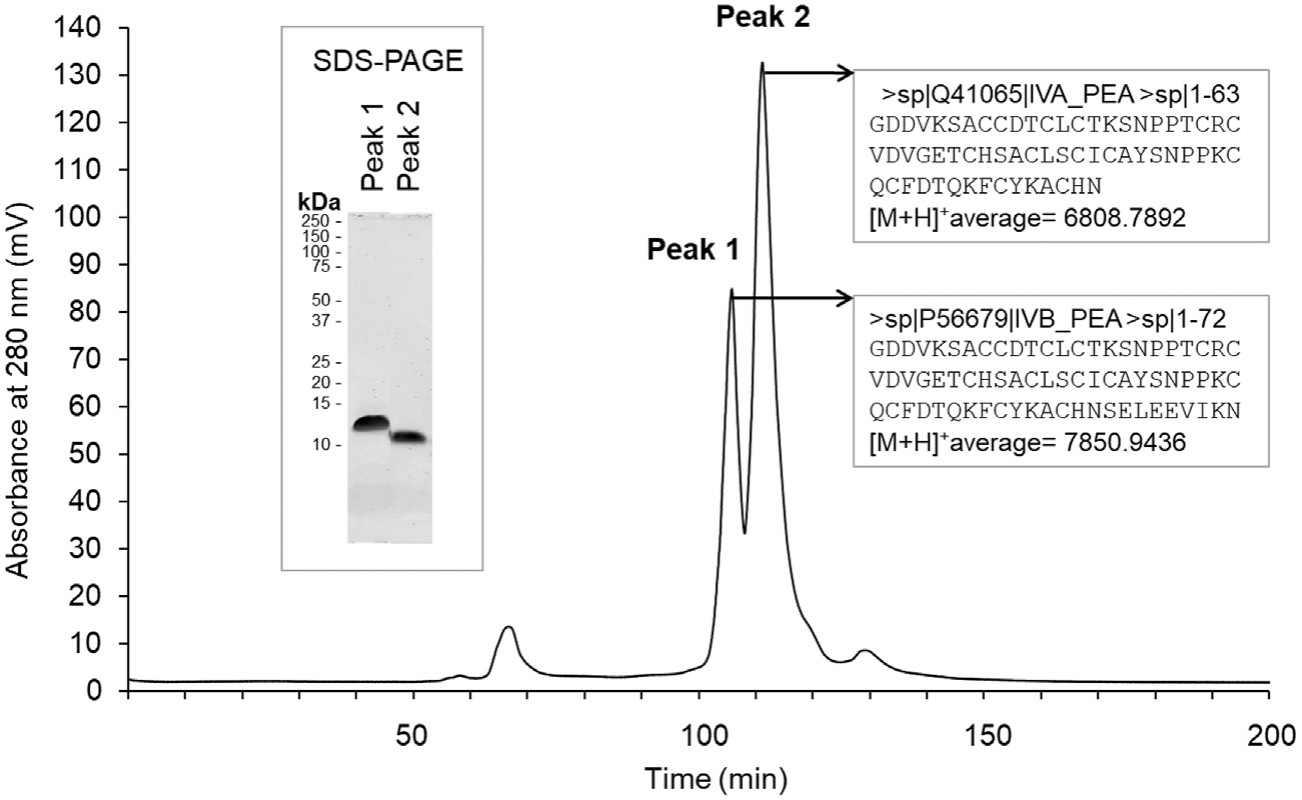
HPLC and characterization of trypsin-agarose eluted samples. The 2 major peaks were analyzed by SDS-PAGE (left insert) and mass spectrometry (right insert), as described in Materials and Methods section.

**Figure 4:**
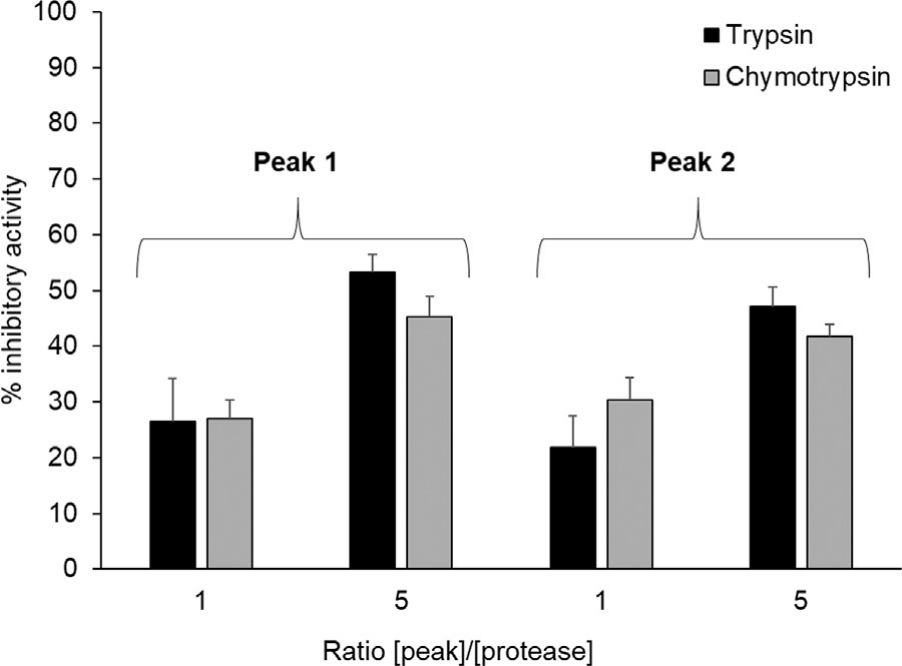
Inhibitory activities of HPLC peak 1 and peak 2. Inhibitory activity of peak 1 and peak 2 was assessed against bovine trypsin (black boxes) and bovine chymotrypsin (gray boxes) using a ratio [peak]/[protease] of 1 or 5.

Considering that BBI is a major inhibitor of trypsin-like proteases, BBI was purified using trypsin-agarose affinity chromatography followed by size-exclusion chromatography, as described in Materials and Methods. The chromatographic profile from size exclusion chromatography revealed the presence of 2 major peaks (retention time of 105 and 115 min). These 2 peaks exhibit an apparent molecular weight of 13 kDa (Peak 1) and 12 kDa (Peak 2) on SDS-PAGE (left insert, Figure 3). Mass spectrometry analysis demonstrated that peak 1 and peak 2 correspond to 2 proteoforms of the Bowman-Birk inhibitor (left insert, Figure 3). The amino acid sequence of peak 2 refers to the native form of the BBI [1–72] (seed trypsin/chymotrypsin inhibitor IVB, P56679) that is further processed into the [1–63] proteoform that lacks 9 amino acid residues at the carboxy-terminal extremity of the protein sequence (right insert, Figure 3). The [M+H]^+^ average was 7,850.9436 and 6,808.7892 for peak 1 and peak 2, respectively. These values correspond to a monomer of each proteoforms. The inhibitory activity of purified BBI proteoforms (peak 1 and peak 2) was analyzed using chromogenic substrates, and bovine trypsin and chymotrypsin as targeted proteases. Figure 4 shows that the 2 proteoforms of BBI exhibit similar inhibitory activity against both bovine trypsin and chymotrypsin.

### Modeling the Interaction Between BBI and Chicken Serine Proteases Named PRSS2 and PRSS3

**Figure 5:**
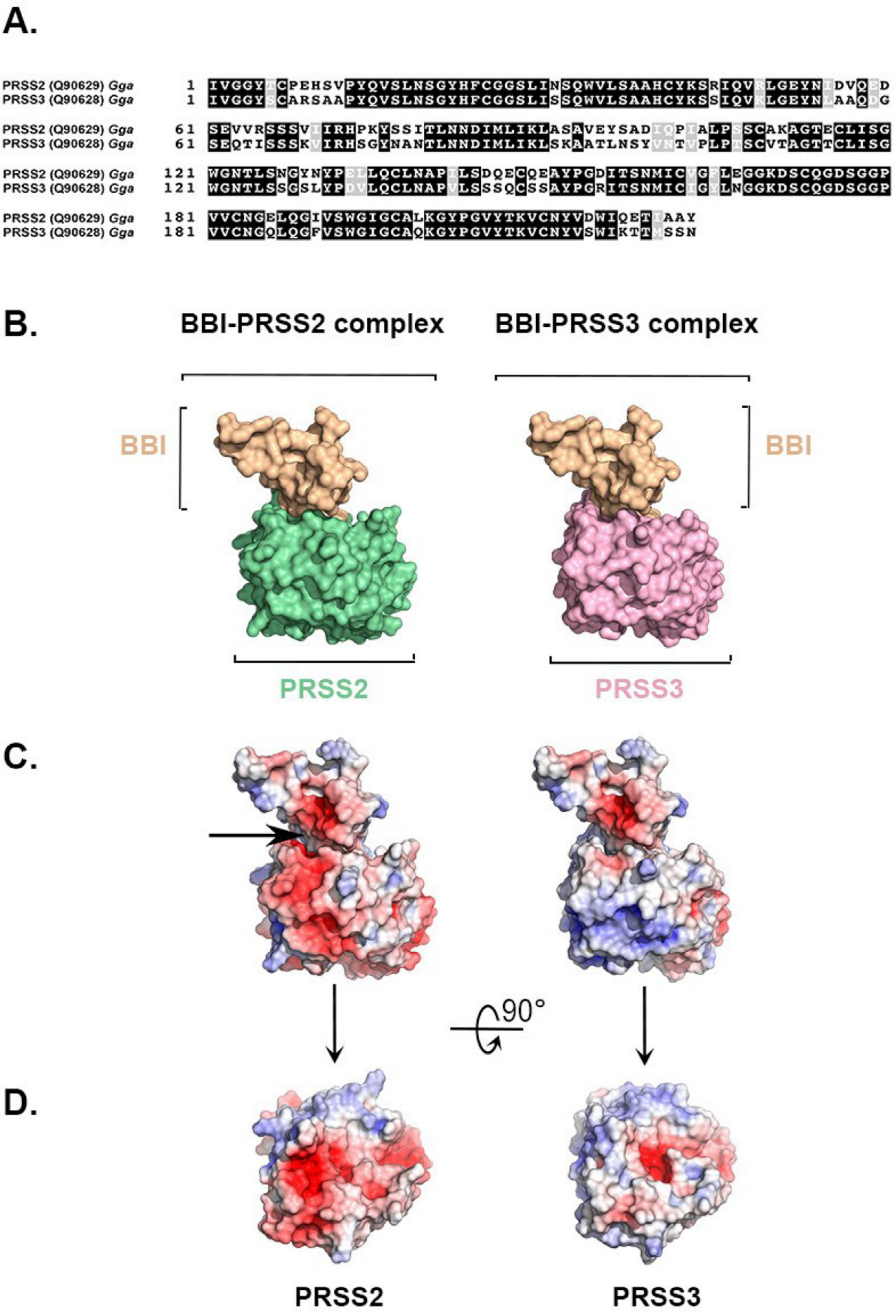
Characterization of PRSS2 and PRSS3, and PRSS2-BBI and PRSS3-BBI complexes. (A) Sequence alignment of chicken PRSS2 and PRSS3 protein sequences. PRSS2 shares 70% sequence identity with PRSS3. Identical residues are represented as white letters in a black box, while similar residues are represented as white letters in a gray box. The alignment was performed using CLUSTAL O (http://www.ebi.ac.uk/Tools/msa/clustalO/) and the printout was created using BOXSHADE software (http://www.ch.embnet.org/software/BOX_form.html). (B) 3D models of BBI-PRSS2 and BBI-PRSS3 complexes (left and right panels, respectively). PRSS2 and PRSS3 models were obtained by comparative modeling using SWISS-MODEL server as described in Materials and Methods section. Protease-BBI complexes were reconstructed by superimposing pea BBI structure (PDB accession code: 1PBI) and either PRSS2 or PRRS3 model onto the inhibitor or protease moiety of the X-ray structure of the soybean BBI-trypsin complex (PDB accession code: 1D6R). (C) Solvent-accessible surface of each partner of the protease-BBI complex. BBI, PRRS2, and PRSS3 are colored light orange, light green, and light magenta, respectively). The figure illustrates the molecular surface of each molecule of the complex (same orientation as in panel B), colored according to values of electrostatic potential values, ranging from −5 kT/e (red) to +5 kT/e (blue), calculated at pH 7.0. In the BBI-PRSS2 complex, a negative patch of the protease is very close to another negative region of BBI (black arrow), suggesting that electrostatic repulsion could prevent or alter the formation of BBI-PRSS2 complex. D. Top view of the BBI binding site in PRSS2 (left) or PRSS3 (right). The figure displays the repartition difference of negative and positive charges, which might influence BBI binding and/or inhibitor affinity with each protease. The figure was prepared with PYMOL software (Schrödinger, 2021).

BBI is known to interact with trypsin-like and chymotrypsin-like proteases. The trypsin-like proteases identified in the 34 kDa band are PRSS2 and PRSS3 that shares 74% protein sequence identity (Figure 5A). Because PRSS3 is assumed to be specifically expressed and secreted in response to BBI diet, we explored the interaction between PRSS3 and BBI as compared with PRSS2-BBI interaction, by building models of protease-BBI complexes (Figure 5B). Results show that the charge distribution in the inhibitor binding site region varies markedly depending on the protease (Figure 5C). This difference may be explained by the fact that PRSS2 is very acidic (calculated pI = 4.68) in contrast to PRSS3 that is a cationic enzyme (calculated pI = 8.58), while BBI pI is slightly acidic (pI = 5.5 and 6.8 for [1–63] and [1–72] proteoforms, respectively, Figure 3). Therefore, the PRSS2 molecular surface in the area interacting with pea BBI is mostly negatively charged (red patches in Figure 5D, left panel) while the corresponding region in PRSS3 has only a negative spot due to Asp189 (red patch in Figure 5D, right panel). Based on these models, we expect a negative-negative charge conflict at the interface of pea BBI with PRSS2 (Figure 5C, left panel). Conversely, no charge incompatibilities were observed between the 2 partners of the PRSS3-BBI complex (Figure 5C, right panel). Overall, these results suggest that pea BBI binding to PRSS3 is probably favored over the PRSS2 protease.

## DISCUSSION

Bowman-Birk inhibitors are small cysteine-rich inhibitors that are primarily found in plants (Mello et al., 2003) in particular in the seeds of legumes. This double-headed inhibitor possesses 2 inhibitory sites, 1 for trypsin-like proteases and 1 for chymotrypsin-like proteases (Birk, 1985, 1996). Functions of BBIs encompass regulation of endogenous seed proteinases, storage of sulfur amino acids, and plant defense (Mello et al., 2003). They have been shown to be expressed in response to water deficiency or drought (Dramé et al., 2013; Malefo et al., 2020) and may also be involved in seed development (Qu et al., 2003). Many articles suggest that BBI is a defensive molecule adapted to respond to plant injuries induced by insect larvae or pathogens (Casaretto and Corcuera, 1995; Qu et al., 2003; Padul et al., 2012; Tan et al., 2013). BBI expected mechanism of action relies on the inhibition of digestive proteases of insects/predators and microbial proteases of pathogens. The inhibitory activity of plant BBI on digestive proteases is supposed to impair the proper digestion of consumers/predators, thus limiting the availability of free amino acids that is necessary for animal growth and development (Mendonça et al., 2020). To counteract the deleterious effect of plant BBI, some insects have developed an adaptive response by expressing digestive proteases that resist BBI inhibitory activity (Paulillo et al., 2000), which nicely illustrates coadaptation between plants and insects during evolution.

Considering that pea can also be a valuable source of proteins for vertebrates including humans, pigs or chickens, there is an urgent need to investigate the impact of the consumption of pea containing active BBI, on the function and proteolytic activities of host digestive proteases. In chickens, BBI from pea and other plant feedstuffs including soybean, is a major antinutritional factor that is associated with delayed and altered protein digestion (Toghyani et al., 2020). To limit this well-known deleterious effect of BBI on host digestive processes, some authors have proposed to select plants that express natural BBI variant(s) with impaired protease inhibitory activity (Clementea and Domoney, 2006).

By comparing the proteolytic profiles of digestive secretions collected from chickens fed 4 protein-rich feedstuffs including pea, we confirmed that active BBI from pea deeply impairs proteolytic activities in chicken intestinal lumen, as compared with other conventional feedstuffs (soybean, corn, or rapeseed, Figure 1). In samples corresponding to the pea diet, we observed a major proteolytic band appearing around 34 kDa that increases in activity from the gizzard to the ileum (Figure 1). In parallel, we showed that pea (*Pisum sativum*) diet contains BBI (Figure 2) that was identified as 2 active proteoforms (Figure 3). The [1–72] proteoform corresponds to the native sequence of BBI while [1–63] proteoform that is predominant, lacks 9 carboxy-terminal amino acids (Figure 3). Both proteoforms have been reported in literature (Domoney et al., 1995). The trimmed [1–63] proteoform has been described to specifically appear during desiccation, and was shown to exhibit increased affinity for trypsin (Domoney et al., 1995). In our experiments, both proteoforms exhibit comparable inhibitory activity against bovine trypsin and chymotrypsin. The identification of proteases composing the digestion-resistant band of 34 kDa revealed the presence of 3 proteases, that are chymotrypsinogen 2-like (**CTRB2**), trypsin II-P39 (**PRSS2**), and trypsin I-P38 (**PRSS3**) (Figure 2), of similar theoretical molecular masses (23–27 kDa). The discrepancy between the molecular masses that is detected on acrylamide gel (34 kDa) vs. theoretical molecular mass for these molecules, is likely explained by the nonreducing conditions that are required for gelatin zymography. Indeed, all 3 proteases were mostly abundant in a 24 kDa band using reducing conditions after boiling (Recoules et al., 2017), which is consistent with their theoretical molecular masses. Considering the ability of BBI to inhibit trypsin-like and chymotrypsin-like proteases, well known CTRB2 and PRSS2 digestive enzymes are likely to form a protease-inhibitor complex with BBI. In contrast, the digestive PRSS3 protease is assumed to have evolved to resist BBI inhibitory activity. Indeed, in human species, PRSS3 protein (also named mesotrypsin) has been shown to cleave diet-derived BBI and inactivate it (Szmola et al., 2003; Alloy et al., 2015).

The presence of denaturing agents such as SDS in the SDS-PAGE gel is supposed to dissociate CTR2-BBI, PRSS3-BBI, and PRSS2-BBI noncovalent complexes that consequently, recover in a single 23 to 27 kDa band. The presence of CPA5 carboxypeptidase in this band remains to be investigated but it might correspond to a cleaved form of chicken CPA5, as its theoretical molecular mass is 45 kDa. Because carboxypeptidases do not interact with Bowman-Birk inhibitors, we will not discuss about a hypothetical CPA5-BBI complex. After renaturation of proteases that are embedded in the acrylamide gel (using Triton X-100) and incubation in activation buffer at 41°C (physiological temperature of chickens), proteolytic activities were detected. Results indicated that maximal activities of these proteases are visible in the ileum segment. In humans, PRSS2, PRSS3 and CTR2 proteases were shown to be essentially expressed in the pancreas (Fagerberg et al., 2014), prior to secretion in the intestinal lumen.

The analysis of the electrostatic potential of chicken PRSS2 and PRSS3 together with that of PRSS2-BBI and PRSS3-BBI complexes (Figure 5B–D) reveals that the interaction of PRSS3 with BBI may be more favorable than that of PRSS2 with BBI, where some small electrostatic conflicts are likely to occur. Indeed, the difference in charge distribution in the region interacting with BBI (Figure 5D) is expected to strongly influence inhibitor binding, as previously suggested for human PRSS3 (mesotrypsin) (Katona et al., 2002), a trypsin-like enzyme known to resist protease inhibitors. Nevertheless, to confirm this hypothesis that is predicted from the analysis of PRSS2/PRSS3-BBI models, it would be necessary to perform kinetic studies (affinity constants) with purified or recombinant chicken PRSS2 and PRSS3 proteases.

Chicken PRSS3 is supposed to act as a “suicide protease” to prevent inhibition of PRSS2 by BBI and allow PRSS2 to digest other proteins of the diet. In humans, mesotrypsin (PRSS3 protease) contained in the pancreatic juice was demonstrated to resist natural protease inhibitors (Rinderknecht et al., 1984). Compared to other human trypsins and to bovine trypsin, mesotrypsin possesses 2 amino acid residues, Serine 39 and Arginine 193 that are sufficient to confer resistance to inhibition by protease inhibitors (Katona et al., 2002; Szmola et al., 2003; Salameh et al., 2008, 2012). In addition to the amino acid residues, Lysine 74 and Aspartate 97 are necessary to induce the full capability to mesotrypsin to degrade bovine pancreatic trypsin inhibitor and amyloid precursor protein Kunitz protease inhibitor that are 2 small-sized protease inhibitors (Alloy et al., 2015). Although gene name for mesotrypsin is PRSS3, amino acids occupying positions 39, 74, 97, and 193 in chicken PRSS2 and PRSS3 are similar to those found in bovine trypsin, but not to those found in human PRSS3/mesotrypsin. This observation suggests that chicken PRSS3 is likely to act a targeted protease for BBI but there is to date no evidence that PRSS3 can inactivate BBI by proteolytic degradation similar to human PRSS3 (Alloy et al., 2015), or resist BBI inhibition as it was previously demonstrated in rat species (Holm et al., 1991).

In conclusion, we have demonstrated that the active BBI contained in the pea diet affects endogenous proteolytic activities in the chicken digestive tract. The presence of active BBI in the intestine is thought to induce the expression of Trypsin I-P36 (PRSS3) that is secreted in the intestinal lumen, and binds BBI. We hypothesize that the formation of Trypsin I-P36/BBI complex decreases the availability of BBI active molecules in the lumen, thus enabling the well-known Trypsin II-P39 (PRSS2) protease to proceed with the digestion of other diet-derived proteins.

In future experiments, it might be interesting to explore the expression of these proteases in the pancreas of animals exposed to pea (Holm et al., 1988; Reseland et al., 1996), to confirm the specific expression of PRSS3 in response to this BBI-containing diet and more generally to all diets containing active trypsin inhibitors. Indeed, we suggest that the presence of PRSS3 in the lumen of intestinal tract could indicate a digestive impairment and be further used as a biomarker of intestinal stress. Several articles have reported an increased expression of PRSS3 (mesotrypsin) in various human digestive pathologies, including colon adenocarcinoma (Zhang et al., 2021), gastric cancer (Wang et al., 2019), irritable bowel syndrome (Rolland-Fourcade et al., 2017), or pancreatitis (Szmola et al., 2003; Toldi et al., 2020). Thus, it is expected that chickens fed with diets containing trypsin inhibitors might overexpress PRSS3 that could have adverse effects on the physiology of the intestinal tract (degradation) if its activity is not regulated. This study highlights the necessity to fully characterize chicken diets, especially new emerging feedstuffs, including insect-derived feedstuffs (Zsedely et al., 2022; Facey et al., 2023; Nieto et al., 2023), to ensure that they do not contain active trypsin inhibitors or other antinutritional factors that could affect animal performances, and be deleterious for animals in the medium/long term.
